# Non-Invasive Prenatal Testing Using Cell Free DNA in Maternal Plasma: Recent Developments and Future Prospects

**DOI:** 10.3390/jcm3020537

**Published:** 2014-05-21

**Authors:** Peter Benn

**Affiliations:** Department of Genetics and Developmental Biology, Human Genetics Laboratory, University of Connecticut Health Center, 263 Farmington Avenue, Farmington, CT 06030-3808, USA; E-Mail: benn@nso1.uchc.edu; Tel.: +1-860-679-3614

**Keywords:** prenatal screening, prenatal diagnosis, non-invasive prenatal testing, cell-free DNA, aneuploidy, monogenic disorders, sequencing, Down syndrome, sex chromosome abnormalities

## Abstract

Recent advances in molecular genetic technologies have facilitated non-invasive prenatal testing (NIPT) through the analysis of cell-free fetal DNA in maternal plasma. NIPT can be used to identify monogenic disorders including the identification of autosomal recessive disorders where the maternally inherited mutation needs to be identified in the presence of an excess of maternal DNA that contains the same mutation. In the future, simultaneous screening for multiple monogenic disorders is anticipated. Several NIPT methods have been developed to screen for trisomy. These have been shown to be effective for fetal trisomy 21, 18 and 13. Although the testing has been extended to sex chromosome aneuploidy, robust estimates of the efficacy are not yet available and maternal mosaicism for gain or loss of an X-chromosome needs to be considered. Using methods based on the analysis of single nucleotide polymorphisms, diandric triploidy can be identified. NIPT is being developed to identify a number of microdeletion syndromes including α-globin gene deletion. NIPT is a profoundly important development in prenatal care that is substantially advancing the individual patient and public health benefits achieved through conventional prenatal screening and diagnosis.

## 1. Introduction

In 1997, Lo *et al.* reported that plasma from pregnant women carrying male fetuses contained cell free DNA (cf-DNA) derived from the Y-chromosome [1]. This was quickly followed by reports that this cf-DNA could be used for accurately determining fetal sex and Rhesus blood group type [2,3,4]. It was subsequently established that the “fetal” component of cf-DNA was actually primarily derived from trophoblasts [5] and had a very short half-life so there was no concern that analysis of this material might reflect a past pregnancy [6,7,8]. The screening and diagnostic potential has been widely recognized and there have been extensive research efforts and clinical trials to develop effective and accurate non-invasive prenatal testing (NIPT). In 2011, the first tests to detect fetal Down syndrome were launched in China and the USA, quickly followed by tests for additional fetal aneuploidies [9]. Based on business reports, it is likely that in the USA alone, in excess of 500,000 NIPT studies on women at high risk for fetal aneuploidy were performed in 2013. The testing is widely expected to be extended to women with low a priori risk, additional major chromosome imbalances, sub-microscopic copy number variation, and various monogenic disorders. NIPT will therefore continue to rapidly expand both in availability and scope.

In this paper, I review latest developments in this rapidly evolving testing and consider future prospects.

## 2. Monogenic Disorders

### 2.1. Current Approaches

#### 2.1.1. Paternally Inherited Autosomal Dominant and *De Novo* Mutation

For disorders that are autosomal dominant with a known paternal mutation, NIPT is based on the detection or exclusion of the paternal mutation in the cf-DNA. This approach has been used in the diagnosis of Huntington’s disease [10,11]; myotonic dystrophy [12] and early onset primary dystonia I [13]. Two of these disorders are associated with trinucleotide repeat expansions that could be difficult to detect when parents share similar allele sizes or where the paternal allele is very large. To resolve this, the detection of closely linked polymorphic regions has been used [14]. A major application of the approach of detecting paternal alleles lies in the prenatal detection of fetal blood group antigens, notably Rhesus-D genotyping, to avoid fetal hemolytic disease. This is reviewed elsewhere [15]. Detection of a fetus with an autosomal dominant disorder with a maternally inherited mutation is much more technically difficult because the fetal genotype in the cf-DNA needs to be identified in the presence of an excess maternal DNA (see below).

There are some autosomal dominant disorders where a new mutation is relatively common and the detection of the mutation in cf-DNA can provide a diagnosis. One such example is achondroplasia where a single mutation in the *FGFR3* gene, c.1138G > A (p.Gly380Arg), accounts for 98% of all cases [16]. Ultrasound findings can sometimes be suggestive of achondroplasia and a non-invasive test that looks specifically for this mutation in cf-DNA can be carried out [17,18]. Thanatophoric dysplasia, also attributable to mutations in *FGFR3*, can similarly be non-invasively diagnosed by looking for the common *de novo* mutations [19]. The choice of cf-DNA testing verses conventional invasive testing may depend on the other skeletal dysplasias that may under consideration in the differential diagnosis because, currently, not all of them will be amenable to a non-invasive diagnosis.

#### 2.1.2. Autosomal Recessive

When both parents are carriers for an autosomal recessive disorder, determining that a fetus is unaffected can be carried out by excluding the paternal mutation in the maternal cf-DNA. This can be carried out relatively easily if the paternal chromosome mutation allele differs from the maternal allele (*i.e.*, if the fetus is potentially a compound heterozygote) or there are closely linked polymorphisms that allow unambiguous identification of the allele that was inherited from the father. The approach has been used for a variety of conditions (reviewed by Bustamente-Aragones *et al.* [20] also [21]). When both parents are carriers for the same mutation or it is otherwise necessary to establish the presence or absence of a particular maternal allele in the fetus, again, there is the significant challenge of characterizing the fetal genotype against a background of a large excess of maternal DNA.

A solution to this difficulty is to quantify the relative numbers of the alleles present in the cf-DNA and establish that there is a statistically significant excess of one type over another, consistent with a presence of one of the two mutations being present in the fetus [22]. As with aneuploidy detection, high numbers are required to detect the marginal difference in counts. Two technical methods have been employed to achieve this, digital PCR and sequencing [23]. A high fetal fraction is advantageous [24] and target enrichment of the region of interest [25] improves the efficacy of these approaches. The approach has recently been illustrated in a prenatal diagnosis of a fetus with the autosomal disorder methylmalonic acidemia [26] where the mother and father were both carriers for the same mutation. Digital PCR droplet technology was used to quantify the fetal DNA fraction (using counts of paternally inherited SNPs) and also to count the mutation and wild type fragments. Consistent with an affected pregnancy, a statistically significant excess of DNA fragments with the mutation was found. SNPs closely linked to the mutation provided additional support for the diagnosis.

#### 2.1.3. Sex-linked or Sex Limited Conditions

A sex-linked or sex-limited genetic disorder can frequently be excluded early in pregnancy simply by establishing that the fetus is not of the at-risk gender. The detection of cf-DNA derived from the Y-chromosome provides a highly accurate determination of fetal sex from as early as 7 weeks gestational age [27,28] which is earlier than gender can be reliably determined by ultrasound. Additional diagnostic testing can then be limited to only those at-risk cases. This practical approach has been used for a broad range of X-linked disorders such as hemophilia and Duchene muscular dystrophy [11]. A serious concern with NIPT applied to sex assessment is its use for non-medical purposes. This is of particular importance in countries where there are social and economic factors favoring having children of a particular sex [29,30,31].

### 2.2. Future Prospects, Generalized Approaches

From the previous summary of current NIPT for monogenic disorders, it should be clear that there are two practical limitations that limit widespread application. First, it is highly advantageous to have the father’s genotype known and for this to be identifiably different from the maternal genotype. Second, identification of the fetal maternally inherited allele DNA fragments amongst a large excess of maternal DNA is technically difficult. Overcoming these obstacles could open the door to NIPT for many more at-risk pregnancies.

**Figure 1:**
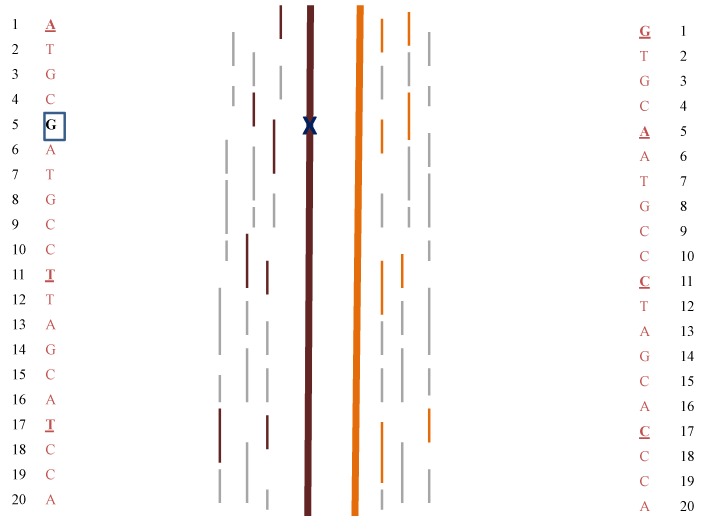
Illustration of SNP analysis in fetal DNA. Dark brown and light brown heavy lines indicate two homologs present in fetal DNA. Thin dark brown and light brown lines represent DNA fragments that may contain SNPs and map uniquely to their corresponding chromosome while thin grey lines are DNA fragments that can map to either homolog. 1–20 refers to representative positions along each chromosome, some of which are the sites of SNPs (bold, underlined bases). Analysis of the fragments present, comparison with the parent’s SNPs, and mapping to reference genome sequences allows construction of the fetal haplotypes. The analysis potentially allows identification of inherited disease causing mutations (e.g., position 5, G boxed) either through identification of the specific fragments with the mutation or through closely linked SNPs.

Lo *et al.* [32] has proposed a generalized method for NIPT that potentially could be used for all monogenic disorders. The fetal haplotype is deduced by evaluating the parents’ genotypes, use of reference genome data, and comparing the relative concentrations of DNA fragments with SNPs (“relative haplotype dosage analysis” or RHDO). The fetal genome is mapped over multiple areas of interest to establish the haplotypes (Figure 1).

**Figure 2:**
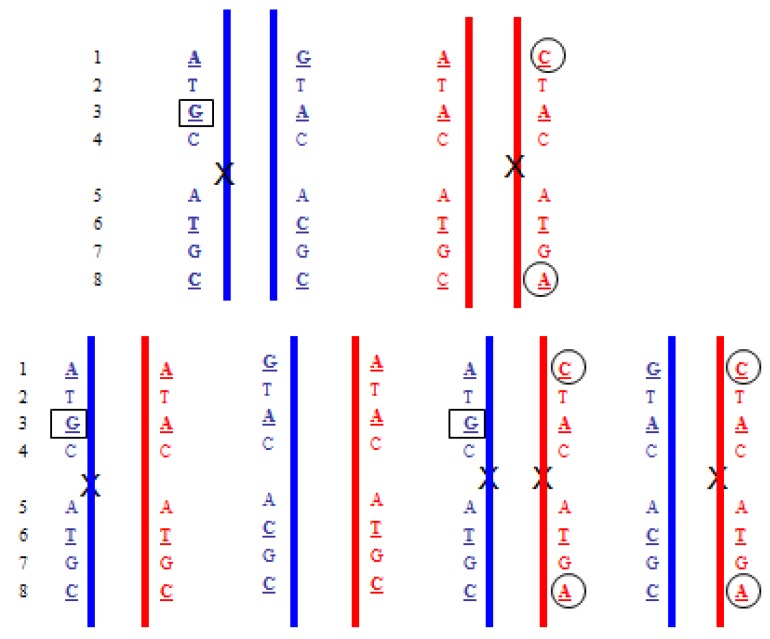
Segregation of an autosomal recessive disorder. Blue indicates paternal chromosomes and red maternal chromosomes. Upper shows the haplotypes for the parents and the lower shows the four different segregation possibilities. X denotes a disease mutation. The identification of the boxed G SNP in the maternal plasma would indicate that the paternal haplotype that carries the mutation was present in the fetus. An excess of the circled C and A SNPs in the maternal plasma (relative to the A and C) would indicate the maternal haplotype with the mutation was present in the fetus.

Figure 2 illustrates how the inheritance of an autosomal recessive disorder would be determined. The method was demonstrated for a pregnancy at risk for β-thalassemia [32]. The method also has the advantage that the paternal genotype might be inferred rather than being determined by direct analysis of a sample from the father. Currently, such an approach that looked at multiple gene regions for many potential disorders would not be cost-effective due to amount of sequencing involved. However, the demonstration illustrates the huge potential for this testing. Indeed, with extensive sequencing it is possible to essentially non-invasively map the entire fetal genome [33,34] and this could include the identification of *de novo* mutation [33], late onset disorders and predispositions.

## 3. Aneuploidy

### 3.1. Methods

Three different testing approaches are currently in use. Shotgun massively parallel sequencing (s-MPS) is based on sequencing and counting of large numbers of unique (single locus) DNA fragments in the plasma and assigning them to the chromosome from which they originated [35,36]. Aneuploidy is evident when there is a relative excess (trisomy) or deficiency (monosomy) for any particular chromosome of interest compared to that expected [35,36,37,38]. Large numbers of fragments need to counted because the difference between aneuploidy and euploidy will be small, especially when the fetal fraction is low. Because there are sequencing biases depending on the GC content of the DNA, adjustments are made to allow for the DNA base composition [39,40]. In principle, it should be possible to apply the testing for the detection of all aneuploidies. In practice, clinical trials have only established the validity for non-mosaic trisomies 21, 18, 13 and, to a lesser extent, monosomy X. Other aneuploidies are extremely rare later in pregnancy and many (for example trisomy 8 and trisomy 9) are often mosaic. Also, it is theoretically possible to apply the method to parts of chromosomes to detect smaller imbalances, including microdeletions and microduplications. To do this, much higher numbers of DNA fragments need to counted which adds to test cost.

A second approach, targeted massively parallel sequencing (t-MPS), includes an additional step that selectively amplifies only those chromosomal regions that are of interest (for example, chromosomes 21, 18 and 13) and then evaluates whether there is an excess for one particular chromosome relative to another [41]. Expanding the scope of the test to look for additional abnormalities is possible but would require a more fundamental redesign of the test than would be the case for s-MPS. An advantage of the t-MPS methodology is a lower sequencing cost (because not all regions need to be sequenced) or alternatively counting higher numbers of DNA fragments that correspond to specific chromosome regions of interest.

**Figure 3:**
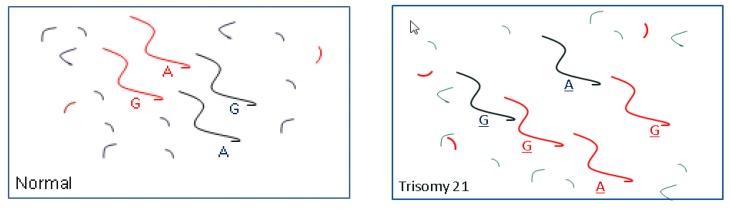
Use of SNPs to detect trisomy 21. Red denotes fetal DNA, black denotes maternal DNA. Left: In this example, for a normal pregnancy, the father is genotyped and known to be GG and the mother GA. The fetus inherited a G allele from the father and A allele from the mother. For a normal pregnancy, the G/A DNA fragment ratio is 1.0 regardless of the fetal fraction percentage. Right: Trisomy 21 is present due to a maternal non-disjunction resulting in a fetal genotype AGG. The G/A fragment ratio will be dependent on the fetal fraction. For example, if the fetal fraction is 20%, the G/A ratio will be approximately ((20% × 2) + (80% × 1))/((20% × 1) + (80% × 1)) = 1.2. The departure from the normal ratio, 1.0, provides evidence for trisomy 21.

The third method that has been developed for NIPT for aneuploidy relies on analyzing SNPs and determining the relative quantitative contributions of maternal and fetal DNA in the plasma. Figure 3 illustrates the general principle for a single SNP that provides evidence for trisomy. One laboratory providing this testing (Natera, Inc., San Carlos, CA, USA) carries out a multiplex PCR amplification on the plasma DNA involving nearly 20,000 SNP sequences in a single reaction [42,43]. This is followed by sequencing to identify which amplified products are present. Each product is evaluated based on the hypothesis that the fetus is monosomic, disomic or trisomic. After considering the positions of the SNPs on the chromosomes and the possibility that there may have been recombination, a maximum likelihood is calculated that the fetus is either normal, aneuploid (chromosome 21, 18, 13 or a sex chromosome) or triploid. The testing can identify regions of fetal chromosome homology that could indicate consanguinity or uniparental disomy. A paternal blood or saliva sample for SNP analysis can improve test performance but it is not essential. The method is expandable to include additional imbalances, including microdeletions and duplications, by identifying sufficient informative SNPs within the region of interest. The method is not limited by counting statistics although, as with other methods, low fetal fraction can be a reason for test failure.

### 3.2. Test Performance: Autosomal Aneuploidy

**Figure 4:**
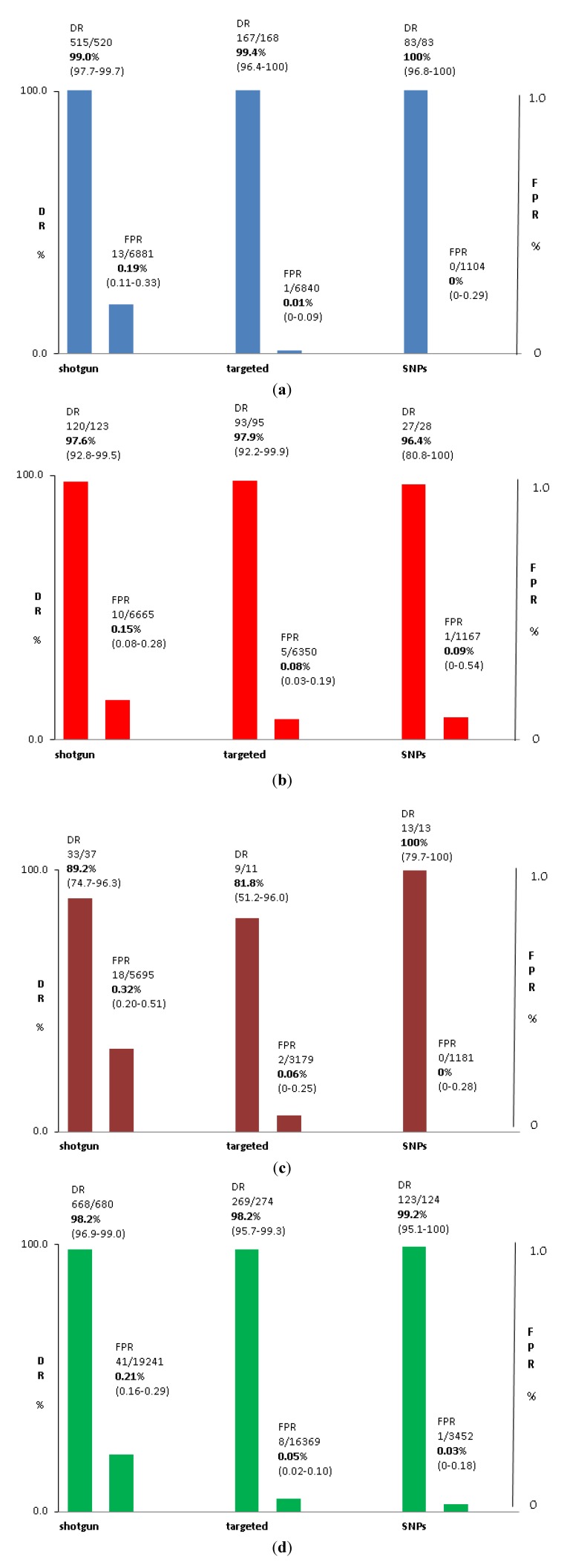
Summary detection rates and false-positive rates for (**a**) Down syndrome; (**b**) trisomy 18; (**c**) trisomy 13; and (**d**) all three trisomies combined for the three NIPT methods.

Table 1 and Figure 4 summarize the data for clinical trials involving the s-MPS approach applied to the detection of trisomy 21, 18 and 13. Prospective trials with incomplete follow-up have been excluded [38,44]. The studies were performed at different laboratories, mostly on high-risk women, and there were variable patient inclusion criteria (advanced maternal age, conventional screen-positive, abnormal ultrasound findings, *etc.*). One study was based on women younger than 35 years old [45] and another involved a total general obstetric population [46]. Also, the cut-offs to define a positive result were variable and there were other differences in test methodologies, including depth of sequencing.

Table 2 and Figure 4 summarize the clinical trial data for trisomy 21, 18 and 13 based on the t-MPS method. The data includes two small studies where the study population included women not selected for their high a priori risk [47,48]. For the low risk women, the test did not appear to perform any differently but, because of the low prevalence of the disorders in this group, extremely large numbers of women would need to be studied to definitely establish this. All testing was performed at a single laboratory (Ariosa, Inc., San Jose, CA, USA).

Performance for SNP method for NIPT is summarized in Table 3 and Figure 4. The data is based on a mixture of patients with high and average a priori risk and is based on testing at a single laboratory (Natera, Inc., San Carlos, CA, USA). Currently available data shows 100% specificity and sensitivity but these need to be interpreted cautiously because rare false-positives and false-negatives can be expected due to biological reasons unrelated to the test performance.

The data in Table 1, Table 2 and Table 3 illustrate that, relative to conventional screening, all methods are extremely effective at detecting and excluding autosomal aneuploidy. As discussed elsewhere in this review, there are approach-specific advantages and disadvantages and some methods may not be optimal or available for certain patients or groups of patients.

**Table 1:** Summary data for NIPT or non-mosaic autosomal aneuploidy using shotgun massively parallel sequencing (s-MPS).

Trial	DS DR	DS FPR	c21 NR	t18 DR	t18 FPR	c18 NR	t13 DR	t13 FPR	c13 NR
Chiu *et al.* [49]	86/86 (100%)	3/146 (2.05%)	11/764 (1.4%)						
Ehrich *et al.* [50]	39/39 (100%)	1/410 (0.24%)	18/467 (3.9%)						
Palomaki *et al.* [51,52]	209/212 (98.6%)	3/1471 (0.20%)	13/1686 (0.8%)	59/59 (100%)	5/1688 (0.30%)	17/1988 (0.9%)	11/12 (91.7%)	16/1688 (0.95%)	17/1988 (0.9%)
Bianchi *et al.* [53]	89/90 (98.9%)	0/410 (0.00%)	16/532 (3.0%) ^a^	35/38 (92.1%)	0/463 (0.00%)	16/532 (3.0%) ^b^	11/14 (78.6%)	0/488 (0.00%)	16/532 (3.0%) ^c^
Liang *et al.* [40]	40/40 (100%)	0/372 (0.00%)	12/435 (2.8%)	14/14 (100%)	0/372 (0.00%)	12/435 (2.8%)	4/4 (100%)	1/408 (0.25%)	12/435 (2.8%)
Song *et al.* [45]	8/8 (100%)	0/1733 (0.00%)	73/1916 (3.8%)	2/2 (100%)	1/1739 (0.01%)	73/1916 (3.8%)	1/1 (100%)	0/1740 (0.00%)	73/1916 (3.8%)
Stumm *et al.* [54]	39/40 (97.5%)	0/430 (0.00%)	32/504 (6.3%)	8/8 (100%)	1/472 (0.21%)	32/504 (6.3%)	5/5 (100%)	0/472 (0.00%)	32/504 (6.3%)
Bianchi *et al.* [46]	5/5 (100%)	6/1909 (0.31%)	17/2042 (0.8%)	2/2 (100%)	3/1905 (0.16%)	17/2042 (0.8%)	1/1 (100%)	1/899 (0.11%)	
Total	99.0%	0.19%	2.30%	97.6%	0.15%	2.3%	89.2%	0.32%	2.8%
(95% CI)	(97.7%–99.7%)	(0.11%–0.33%)	(2.0%–2.7%)	(92.8%–99.5%)	(0.08%–0.28%)	(1.9%–2.6%)	(74.7%–96.3%)	(0.02%–0.51%)	(2.4%–3.3%)

DS, Down syndrome; t18, trisomy 18; t13, trisomy 13. DR, detection rate; FPR, false positive rate; NR, no result due to low fetal fraction or failure due to reasons other than inadequate or ineligible sample for chromosome 21 (c21), 18 (c18) or 13 (c13). ^a^ An additional 7/503 cases were “unclassified” for DS; ^b^ an additional 5/502 were “unclassified” for t18; ^c^ an additional 2/502 were “unclassified” for t13.

**Table 2:** Summary data for NIPT or non-mosaic autosomal aneuploidy using targeted massively parallel sequencing (t-MPS).

Trial	DS DR	DS FPR	c21 NR	t18 DR	t18 FPR	c18 NR	t13 DR	t13 FPR	c13 NR
Ashoor *et al.* [55,56]	50/50 (100%)	0/297 (0.00%)	3/400 (0.8%)	49/50 (98.0%)	0/297 (0.00%)	3/400 (0.8%)	8/10 (80%)	2/1939 (0.05%)	53/2002 (2.6%)
Verweij *et al.* [57]	17/18 (94.4%)	0/486 (0.00%)	16/520 (3.1%)						
Norton *et al.* [58]	81/81 (100%)	1/2888 (0.03%)	148/3228 (4.6%)	37/38 (97.4%)	2/2888 (0.06%)	148/3228 (4.6%)			
Nicolaides *et al.* [47]	8/8 (100%)	0/1939 (0.00%)	100/2049 (4.9%)	2/2 (100%)	2/1929 (0.01%)	100/2049 (4.9%)			
Fairbrother *et al.* [48]	-	0/284 (0.00%)	4/288 (1.4%)	-	0/284 (0.00%)	4/288 (1.4%)	-	0/284 (0.00%)	4/288 (1.4%)
Gil *et al.* [59]	11/11 (100%)	0/946 (0.00%)	48/1005 (4.8%)	5/5 (100%)	1/952 (0.11%)	48/1005 (4.8%)	1/1 (100%)	0/956 (0.00%)	48/1005 (4.8%)
Total	99.4%	0.01%	4.3%	97.9%	0.08%	4.3%	81.8%	0.06%	3.2%
(95% CI)	(96.4%–100%)	(0.00%–0.09%)	(3.8%–4.7%)	(92.2%–99.9%)	(0.03%–0.19%)	(3.9%–4.9%)	(51.2%–96.0%)	(0.00%–0.25%)	(2.6%–3.9%)

DS, Down syndrome; t18, trisomy 18; t13, trisomy 13. DR, detection rate; FPR, false positive rate; NR, no result due to low fetal fraction or failure due to reasons other than inadequate or ineligible sample for chromosome 21 (c21), 18 (c18) or 13 (c13).

**Table 3:** Summary data for NIPT or non-mosaic autosomal aneuploidy SNP based analysis.

Trial	DS DR	DS FPR	c21 NR	t18 DR	t18 FPR	c18 NR	t13 DR	t13 FPR	c13 NR
Nicolaides *et al.* [60]	25/25 (100%)	0/204 (0.00%)	13/242 (5.4%)	3/3 (100%)	0/226 (0.00%)	13/242 (5.4%)	1/1 (100%)	0/228 (0.00%)	13/242 (5.4%)
Pergament *et al.* [61]	58/58 (100%)	0/905 (0.00%)	88/1051 (8.4%)	24/25 (96%)	1/939 (0.11%)	87/1051 (8.3%)	12/12 (100%)	0/953 (0.00%)	86/1051 (8.2%)
Total	100.0%	0.00%	7.7%	96.4%	0.09%	7.6%	100.0%	0.00%	7.7%
(95% CI)	(96.8%–100%)	(0.00%–0.29%)	(6.3%–9.2%)	(80.8%–100%)	(0.00%–0.54%)	(6.3%–9.2%)	(79.7%–100%)	(0.00%–0.28%)	(6.3%–9.2%)

DS, Down syndrome; t18, trisomy 18; t13, trisomy 13. DR, detection rate; FPR, false positive rate; NR, no result due to low fetal fraction or failure due to reasons other than inadequate or ineligible sample for chromosome 21 (c21), 18 (c18) or 13 (c13). NR rate is lower when a sample from the father was available for analysis.

### 3.3. Test Performance: Sex Chromosome Aneuploidy

Robust estimates for the sensitivity and specificity of sex chromosome abnormalities (SCA) only exist for non-mosaic 45,X and even these estimates need to be viewed cautiously. The number of cases in each study is low; there may be ascertainment bias through the preferential inclusion of non-viable cases and those with abnormal serum and ultrasound findings. There is also likely to be incomplete ascertainment of test negative cases because phenotype may not be apparent at birth.

Using the s-MPS method, Bianchi *et al.* [53] observed a 15/20 (75%) detection rate for 45,X with a false-positive rate of 1/462 (0.2%) but with 49/482 (10.2%) samples unclassified (including 4 affected and 45 controls). Similarly, Mazloom *et al.* [62] used the s-MPS method and, for their validation data (excluding training set data), they observed a 17/21 (81%) detection rate for 45,X for a false-positive rate of 1/390 (0.3%) and with 21/411 (5.1%) unclassified (including 3 affected and 18 controls). Using t-MPS, the detection rate was 43/49 (91.5%), the false-positive rate was 0/125 (0%), and the unclassified rate was 5/177 (2.8%) [63]. For the SNP methodology, the detection rate for 45,X was 12/13 (92%) the false-positive rate was 1/954 (0.1%) and the unclassified rate was 87/1051 (8.3%) [43,61].

Although estimates for detection rates and false positive rates for 47,XXY, 47,XXX and 47,XYY are not available, some NIPT providers do provide the testing based on the very limited data that is available. Following a positive result, it not yet possible to counsel with any degree of confidence how likely it is that an SCA is actually present. Consequently, women need to be counseled very carefully prior to the provision of NIPT about this uncertainty because they may be subsequently faced with the dilemma of making a choice between confirmatory invasive testing (with its inherent risk) for a mild disorder, or, accepting a high level of uncertainty for the remainder of pregnancy. Additionally, they need to be informed that, depending on the precise NIPT result and the subsequent work-up to confirm or exclude abnormality, they may learn that they personally have a SCA that is of uncertain clinical significance [64].

### 3.4. Multiple Pregnancies

For twin pregnancies, several laboratories now provide NIPT using DNA counting methods. For monozygotic twins, NIPT should perform substantially equivalently to that achieved for singleton pregnancies. For dizygotic twins and higher multiples NIPT is more problematic because the per fetus fetal fraction may be lower [65,66]. In the situation where one fetus is euploid and the other aneuploid, there is an additional dilution of cf-DNA from the aneuploid pregnancy. For NIPT counting methods, published data on the performance of NIPT is still limited for cases with aneuploid/euploid discordancy [67,68,69,70]. Gil *et al.* [69] concluded that testing in twin pregnancies is feasible but reporting rates will be lower than in singleton pregnancies. Using t-MPS, the test failure rate for twin pregnancies was 13.2% declining to 7.4% after patient redraws [69]. In theory, NIPT methods that identify SNPs should be able to identify multiple fetuses but such testing is not yet offered.

### 3.5. Triploidy

SNP-based methods for NIPT can identify diandric triploidy. Nicholaides *et al.* [71] reported the detection of four such cases detectable because the SNP pattern indicated three copies of chromosome 21, three copies of chromosome18 and three copies of chromosome 13. Although this is a small series, the data from trisomies and controls indicates that such a pattern is highly informative. A similar SNP pattern could potentially be observed with dizygotic twins and therefore ultrasound examination is recommended to help exclude this possibility. The ultrasound examination could miss a vanishing twin but will generally show a thickened or partial molar placenta when diandric triploidy is present. Maternal serum markers may also be helpful because these pregnancies are generally associated with very high hCG with atypical results for other serum markers [72,73]. Identification of partial molar pregnancies is considered to be important because of the risk for persistent gestational trophoblastic neoplasia [74].

In addition, Nicholaides *et al.* [71] also point out that NIPT testing might also provide a strong indication when digynic triploidy is present because the plasma free DNA fetal fraction is often extremely low. Of four cases of digynic triploidy, three had a fetal fraction below the 0.5 percentile, after correcting for maternal weight and gestational age. These levels are too low for NIPT test interpretation but this is another reason why fetal fraction is an important parameter that should routinely be measured and reported. Again, ultrasound can help clarify the situation because digynic triploid pregnancies are associated with a small placenta and severe growth restriction. Usually, the serum markers are also abnormally low [72,73].

### 3.6. Test Failures

Unlike traditional maternal serum screening tests and cytogenetic analyses, failure to obtain a NIPT result is relatively common and this needs to be considered in the clinical management of individual cases. Based on the clinical trials that involved selected cases, no results were obtained in approximately 2% of all tests performed [9].

The most common cause for failing to obtain a result is a low fetal DNA fraction in maternal plasma. A number of laboratories use a fetal fraction cut-off of 4% as the minimum that can be used for test interpretation. Factors that appear to correlate with fetal fraction include maternal weight, gestational age, and serum markers PAPPA and hCG [51,75,76,77,78]. The correlation with serum markers probably reflects an underlying correlation with placental volume. This hypothesis would be consistent with a finding that placental volume is smaller [79] and fetal fraction is lower [80] for pregnancies where fetal trisomy 18, trisomy 13, or dygynic triploidy is present. Based on the means and standard deviations for fetal fraction reported by Rava *et al.* [80] and assuming Gaussian distributions for fetal fraction, it can be crudely estimated that nearly 5% of euploid cases have fetal fraction below 4% but the percentage is substantially higher in trisomy 18, trisomy 13 and 45,X pregnancies.

A low fetal fraction in an initial sample will be associated with a relatively high chance of a similar situation for a second sample. In one study, of 135 cases where there was a redraw due to insufficient fetal DNA, there were 59 (44%) with insufficient fetal DNA in the second sample [77]. This can partly be explained by the inclusion of heavier women and women with small placentas. For at least some women who show a low fetal fraction, it may be appropriate to reconsider invasive testing rather than redrawing for NIPT, especially if there was serum marker or ultrasound evidence suggestive of a high risk for trisomy 18, 13, XO, or digynic triploidy.

### 3.7. Reasons for Discordant Results

In the evaluation of NIPT trials, it has been usual to accept that the results of chromosome analysis of amniotic fluid or chorionic villus samples are correct and constitute “the gold standard”. NIPT results that are discordant are therefore classified as false-positives and false-negatives [81,82]. As experience accumulates, it is becoming clear why some of these discordances arise.

(a) Low fetal fraction and/or insufficient depth of sequencing. For NIPT based on counting DNA fragments (*i.e.*, s-MPS and t-MPS but not SNP based methods), test performance will be strongly dependent on a combination of the fetal fraction and the total numberof DNA fragments counted (*i.e.*, the depth of sequencing) [83]. The effect of fetal fraction was also illustrated by Canick *et al.* [84] who showed that the *z*-values (the test statistic for distinguishing between affected and unaffected cases) for Down syndrome affected pregnancies were relatively close to normal when the fetal fraction was low. Consistent with this, Allen *et al.* [85] described a trisomy 21 NIPT false-negative result that appeared to be attributable to low fetal fraction.
(b) Fetal and placental mosaicism. It is well known from cytogenetic studies that in mosaic cases, the proportion of cells showing each cell type can vary substantially from tissue to tissue and the proportions of cells seen in amniotic fluid cells and chorionic villi specimens can be a poor reflection of that present in individual tissues from the fetus [86]. Sometimes, an abnormal cell line is detected in some tissues but not others. NIPT relies on the analysis of DNA from trophoblasts with results generally reported as either positive or negative. The presence of two cell lines should therefore occasionally cause the NIPT result to appear to be discordant with the cytogenetic result [82]. In the most extreme situation, a cell line may be substantially confined to the placenta (confined placental mosaicism, CPM) with no evidence in other tissues. For some seemingly non-mosaic fetal aneuploidies (for example 45,X), the presence of a second cell line in the placenta may actually be the normal situation in surviving cases [87]. Numerous cases of discrepant NIPT results have now been attributed to CPM. For example, Pan *et al.* [88] described a case in which NIPT was positive for trisomy 21, karyotyping and QF-PCR indicated only two copies of chromosome 21 (but with uniparental disomy), and analysis of placenta showed evidence of a trisomy 21 cell population. Conversely, Wang *et al.* [89] described two cases in which NIPT was negative but where invasive test samples showed apparent non-mosaic trisomy 21 and subsequent analysis of placental biopsies showed mosaicism. Discordancy due to undetected mosaicism can be expected to arise regardless of which NIPT methodology is used.
(c) Maternal chromosome abnormality. Another cause of discrepancy can be attributed to the presence of an abnormal karyotype or cell line in the mother, mistakenly attributed to the fetus. For example, the mother may have a non-mosaic 47,XXX karyotype [64,90], may have a low level constitutional mosaicism involving an autosome [45], a small imbalance [91], or have a malignancy that is karyotypically abnormal [92]. Maternal somatic cell mosaicism is expected to be quite common for loss or gain of an X-chromosome [63]. Wang *et al.* [64] reported evidence for maternal X-chromosome abnormality in 16 of 181 (8.6%) of cases with a NIPT result positive for a SCA. These causes for discordancy confound counting methods for NIPT but the approach based on SNP analyses should potentially be able to distinguish between at least some situations where a maternal mosaicism is present *versus* a fetal abnormality.
(d) Multiple pregnancy and vanishing twin. As noted above, there is less confidence in testing for dizygotic and higher multiple pregnancies. If an aneuploid twin fetus is non-viable and unrecognized (vanishing twin) this could cause a discrepancy between NIPT and the invasive test result or outcome. Since placental tissue can persist long after a fetus is no longer evident and many fetal deaths can be attributed to aneuploidy, it seems likely that this will be increasingly recognized as a cause of false-positive results [44].
(e) Laboratory error. Hopefully, errors in test and reporting procedures are rare and when they do occur corrective actions are implemented to prevent recurrence [44].

## 4. Future Developments in NIPT for Cytogenetic Abnormality

### 4.1. Women at Low Prior Risk

A number of clinical trials have established that NIPT can successfully be offered to women who are not at high a priori risk for aneuploidy [38,45,46,47,48,61]. Because of the low prevalence of aneuploidy in a general population, developing robust estimates for the detection rate and false-positive rates is impractical. However, there is no reason to think that currently formulated NIPT should not perform satisfactorily in a low risk population. A key variable in performance is fetal fraction and Brar *et al.* [93] showed that there was no significant difference in fetal fraction between those at low *versus* those at high prior risk. Furthermore, there was no evidence for major differences in fetal fraction or test performance within high-risk women receiving NIPT for trisomy 21 when sub-grouped by indication [51, Supplemental data].

There is an expectation that providing NIPT to women with low prior risk will be associated with a lower positive predictive value, *i.e.*, more of the NIPT positive results for low risk women will be false-positives [94,95,96]. This expectation assumes that the test detection rate and false-positive rate is substantially independent of the prior risk. If most false-positive and false-negative results are due to random factors such as lab error or inadequate fetal fraction, this assumption is reasonable. However, it is not necessarily true if most discordant results are due to fetal/placental mosaicism, twins, or maternal somatic mosaicism, each of which may be age dependent and therefore have a lower incidence in low risk women. It is therefore possible that NIPT may actually have a somewhat better positive predictive value for low risk women that previously suggested. Even under the conservative assumption that the performance of the test is the same for all women, NIPT would still provide extremely powerful screening when applied to low risk women. For example, for a woman with a very low prior risk of 1:1000, a positive NIPT that has a detection rate of 99.3% and a false-positive rate of 0.16% will mean that the patient has a very high, greater than one in three, chance of an affected pregnancy. The requirement for confirmatory invasive testing for all NIPT positive patients is now firmly embedded in professional guidelines for this testing [97,98,99,100]. The lower positive predictive value should not therefore be held as reason to withhold NIPT from low risk women.

A more substantial practical issue is cost. Most economic analyses have justified the use of NIPT for women at high risk for fetal Down syndrome based on a comparison of NIPT with established screening modalities [51,101,102,103,104]. Before concluding the NIPT is not cost effective for lower risk women, a full assessment needs to consider all the chromosome abnormalities detectable, non-medical costs, and recognize that there are also substantial intangible benefits associated with earlier, safer, and less stressful testing [104].

It also has to be recognized that traditional aneuploidy screening modalities offer the advantage of the detection of additional fetal abnormalities and pregnancy conditions not identifiable through NIPT. Various contingent test protocols have been suggested that would allow women to receive serum and ultrasound marker screening with proportions of women having access to NIPT [105,106,107]. Protocols are likely to vary according to local availability of the tests, economic conditions, and patient preferences. Hopefully, NIPT will become increasingly available to more women while still preserving the additional benefits of traditional screening.

### 4.2. Other Aneuploidy and Chromosome Imbalances

Geux *et al.* [108] have proposed an s-MPS test enhancement with somewhat increased depth of sequencing and GC-bias removal which they claim has a better spatial resolution and molecular precision than karyotyping. Chen *et al.* have devised a modified algorithm for assessing the data to identify partial chromosome imbalances [109]. Efforts to enrich the fetal fraction in cf-DNA could also lead to improved testing [25,110,111,112]. After excluding trisomy 21, 18, 13 and 45,X, most other chromosome abnormalities are rare later in pregnancy and it will be challenging to include these additional abnormalities while keeping the overall false-positive rate low. However, development of the testing to identify early non-viable pregnancies with aneuploidy [9], detection of mosaicism, microdeletion syndromes, and translocations are all areas where enhancement of NIPT for cytogenetic abnormality can be envisaged.

### 4.3. Mosaicism

Because two or more cell lines are present in approximately 14% of abnormal amniotic fluid [113] and 45% of abnormal CVS studies [114], the ability to identify mosaicism is important. Current NIPT tests are mostly based on clinical trials where mosaic cases were excluded. Based on the limited data available for cases where an autosomal or sex chromosome abnormality was known to co-exist with a normal fetal cell line, about half the cases were identified by NIPT [53,67]. In theory, when the fetal fraction is high but test result is intermediate, the results could be indicative of mosaicism or a partial duplication/deletion [83]. Presentation of results in a format that indicates a probability of abnormality, rather than a categorical positive or negative, might aid in the identification of mosaic cases. More data is needed to evaluate when it would be worthwhile to suggest additional invasive testing in cases with intermediate NIPT findings.

### 4.4. Microdeletions and Microduplications

Using the NIPT counting methods, small duplications and deletions (or copy number variations, CNVs) can potentially be detected through NIPT provided sufficient DNA fragments are counted [83]. Modeling indicates that both the size of the region and the depth of sequencing are important and that efficacy of detecting duplications and deletions should be similar [83]. Using deeper sequencing, a number of examples of duplications and deletions that have now been described [115,116,117,118]. At least one laboratory currently offers NIPT using the counting method for several microdeletion syndromes but data on their depth of sequencing, validation data, detection rate, and false-positive rate is currently not available [119]. For methods based on SNPs, the detection of a microdeletion or duplication relies on the identification of sufficient informative SNPs within the region of interest [120].

An important special example for microdeletion testing lies in the ability to test for α-thalassemia. In Southeast Asia, α-thalassemia arising from the homozygous deletion of the two tandem copies of the α-globin genes on both chromosomes (-α/-α, -α/-α) is associated with hydrops fetalis. Because the tandem -α/-α globin deletion has a high frequency (up to 15% in some regions), diagnosing the disorder has been a common indication for invasive prenatal testing. The size of the deletion is less than 1.5 Mb.

One approach is to identify SNPs within the commonly deleted gene segment and deduce whether the paternal normal or deleted chromosome was inherited by the fetus [121]. As previously discussed, Lo *et al.* [32] has demonstrated that any disorder could potentially be non-invasively diagnosed using a maternal plasma cf-DNA deep sequencing strategy that takes advantage of the relative haploid dosage of closely linked SNP markers. In a subsequent proof of principle study, Lam *et al.* [122] performed a target enrichment step for the α-gene locus region followed by relative haploid dosage analysis of the closely linked SNPs. The advantage of the target enrichment is much lower sequencing costs. Another approach to the diagnosis involves target enrichment and then directly measuring the copy number of the α-globin segment, relative to controls. Ge *et al*. [123] noted that after the enrichment, the concentration of cf-DNA in each sample does differ considerably for the different fragments analyzed. However, using appropriate controls, they developed an algorithm to assess the copy number. They successfully applied this in the non-invasive detection of one fetus with homozygous deletion, two fetuses with heterozygous deletion, and an additional two cases with normal α-globin gene copy number. Ge *et al.* [123] point out that the same strategy could be used for other pathogenic copy number variants.

Taken together, these reports indicate that there is substantial potential to develop safe and effective non-invasive screening tests for microdeletions and microduplications and substantially reduce the need for invasive tests for these disorders.

### 4.5. Treanslocations

**Figure 5:**
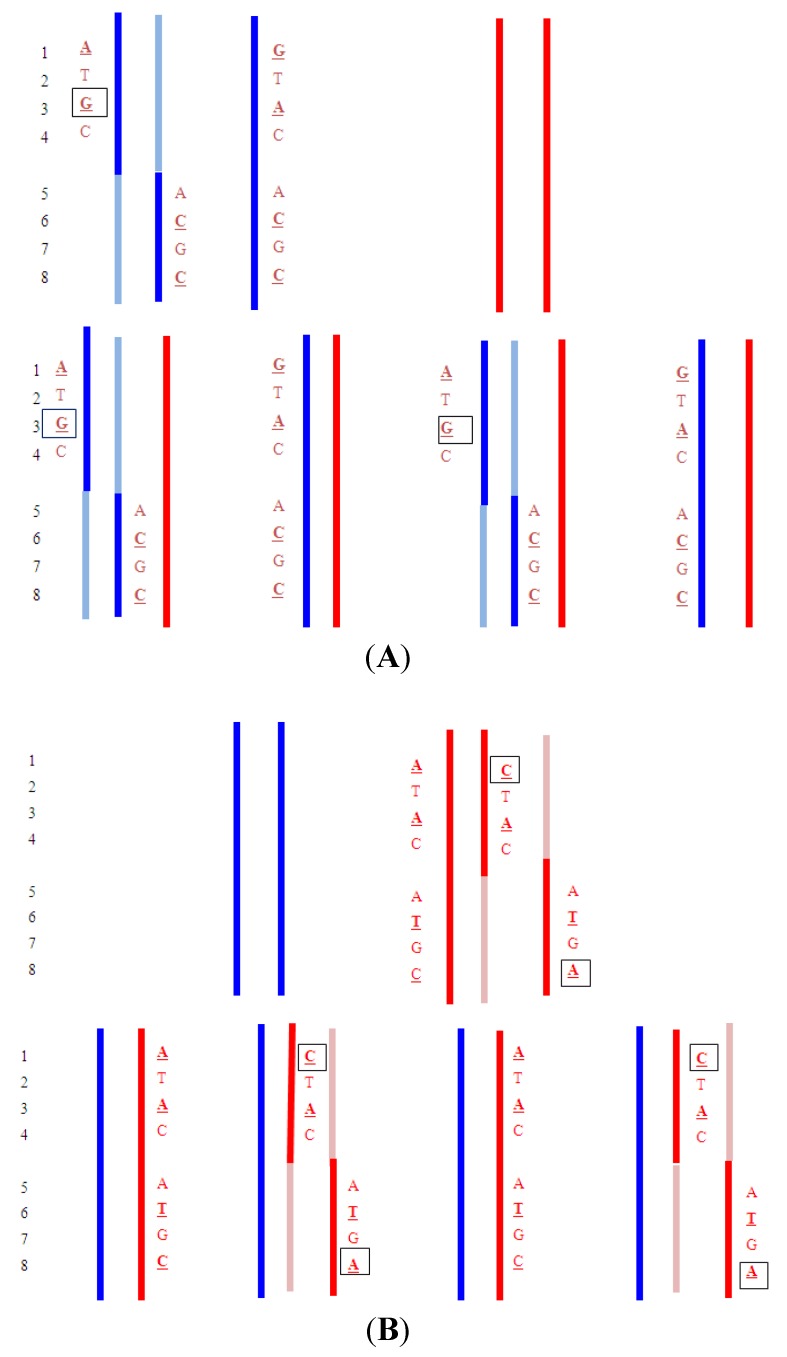
(**A**) Detection of a paternally inherited balanced translocation; (**B**) Detection of a maternally inherited balanced translocation.

When a balanced translocation is known to be segregating in a family, the presence or absence in a fetus could potentially be established non-invasively. The strategy would be to sequence large DNA fragments from a carrier parent such that junction sequence information and SNPs close to the breakpoints were mapped. The maternal plasma cf-DNA would then be analyzed for the presence or absence of these junction sequences and SNPs (Figure 5A, Figure 5B). Identifying a *de novo* translocation would be much more challenging but might eventually be achievable by interrogating the sequences for the presence of matching reciprocal exchange junction fragments.

- Upper: Paternal reciprocal translocation between two chromosomes (light blue and dark blue). The normal homolog (solid dark blue chromosome) and the maternal copies of the same chromosomes (red) are shown. Haplotypes for the blue chromosome adjacent to the breakpoint are shown. Underlined bases are polymorphic, and boxed are informative. Lower: The four possible segregation products. The detection of the DNA fragments with 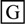 indicates that the translocation chromosome was inherited. In practice, multiple linked SNPs would be used to be certain which chromosome was segregating. Similar analyses can be carried out for the light blue chromosome (haplotypes not shown).
- Upper: Maternal reciprocal translocation between two chromosomes (red and pink). The normal homolog for the red chromosome together with the paternal copies of the same chromosome (blue are shown). Haplotypes for the red chromosome adjacent to the breakpoint are shown. Underlined bases are polymorphic, and boxed are informative. Lower: The four possible segregation products. The detection of an excess of the DNA fragments with 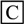 and 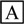 indicate that the translocation chromosomes were inherited. The maternally inherited haplotypes need to be detected in a background of maternal DNA and therefore the testing relies on detecting a relative excess (or deficiency) of particular fragments with the particular polymorphism.

## 5. Conclusions

The development of NIPT is a profoundly important advance in prenatal care. Combined with advances in carrier screening [124], and diagnostic testing, NIPT provides an opportunity to significantly reduce the burden associated with birth defects. Moreover, early identification of affected pregnancies opens the door to fetal therapy [125]. For most women, NIPT will provide early reassurance. There can be little doubt that established prenatal screening and diagnosis, combined with advances in assisted reproductive technology, have reduced fear and allowed women to pursue education and careers before having children. By avoiding the dangers associated with invasive testing [126], NIPT takes these benefits to a new level.

A challenge for the medical community is the pace of NIPT development and clinical introduction. The decision to include a new NIPT is made by the clinician, not by the laboratories, and it is often difficult to separate commercial promotional statements from objective test performance assessments. Tests may be offered before any professional guidelines are available and before peer reviewed publication of validation studies. In this situation, at a minimum, providers of the testing should provide comprehensive details on their web sites so that the utility can be fairly evaluated.

Finally, it should be recognized that there are significant challenges in counseling patients about the highly technical prenatal testing options now available and their implications. It is often necessary to counsel women based on very limited data. Moreover, because of the rarity of many of the disorders, only indirect evidence may be available to evaluate efficacy of new NIPT. For the full medical benefits of the testing to be fully realized, there needs to be additional emphasis on outcome data collection. As a practical matter, if the new testing is to achieve its maximum potential, there also needs to be greater emphasis on the development of patient educational materials that can explain the nature and implications of these new tests.
